# Preparation, characterization and in-vivo efficacy study of glatiramer acetate (GA)-hydrogel-microparticles as novel drug delivery system for GA in RRMS

**DOI:** 10.1038/s41598-022-26640-x

**Published:** 2022-12-21

**Authors:** Naghmeh Hadidi, Gholamreza Pazuki

**Affiliations:** 1 Department of Clinical Research and EM Microscope, Pasteur Institute of Iran (PII), Tehran, Iran; 2 Department of Chemical Engineering, Amirkabir University of Technology, Tehran, Iran

**Keywords:** Biotechnology, Drug discovery, Immunology, Neuroscience, Neurology

## Abstract

Relapsing Remitting Multiple Sclerosis is a chronic Central nervous system autoimmune disease. There is no absolute treatment for MS and the available remedies are called disease modifying therapies (DMTs). Glatiramer acetate (GA) is one of the FDA approved DMTs. Currently, injection-site problems and unfavorable daily injection are the most common milestones in administration of GA. So that, the design of improved drug delivery systems with sustained release profile seem necessary and helpful in order to minimize GA adverse effects and improve patients’ compliance. In this study, we have manufactured a novel chitosan-PLGA (poly (lactic-co-glycolic acid)) hydrogel-microparticles containing GA by double emulsion method. Hydrogel-microparticles’ properties including size, morphology and GA loading were investigated. In-vitro drug release was studied during 30 days. In vivo efficacy of GA-hydrogel-microparticles was evaluated in experimental autoimmune encephalomyelitis (EAE) as an established animal model for MS. Pathological studies were performed through H&E (Hematoxylin and Eosin) staining of brain, spine, liver, skin and kidney tissues. Luxol fast blue staining of brain tissue was also done. The obtained results were applied for safety and efficacy evaluations. GA loading and Entrapment efficiency (EE %) of 60% and 95% were achieved, respectively. In- vitro release studies confirms a sustained release profile for GA-hydrogel-microparticles. Mean clinical scores and mean body weights obtained from EAE animal model for GA-hydrogel-microparticles were compared to the outcomes achieved from conventional Iranian brand-generic injection solution of GA (Copamer^®^, 20 mg/ml). EAE outcomes and pathological studies confirm similar therapeutic efficacy with longer dosing intervals possibility, improved safety through decreased adverse effects and elimination of site injection reactions for GA-hydrogel-microparticles. Further studies on pharmacokinetic and pharmacodynamics in human volunteers are still required to thoroughly examine different aspects of this newly developed GA- hydrogel-microparticles.

## Introduction

Multiple sclerosis (MS) is a chronic autoimmune disease. It may be defined as a neurological dysfunction caused by degeneration of white and gray matter of brain and spinal cord. Most MS patients are women and its prevalence is different from 5 to 80 per 100,000 persons worldwide^1–3^. There are five major types of multiple sclerosis: (1) Benign Multiple Sclerosis, (2) Relapsing–Remitting Multiple Sclerosis (RRMS), (3) Secondary Progressive Multiple Sclerosis (SPMS), (4) Primary Progressive Multiple Sclerosis (PPMS), (5)Progressive Relapsing Multiple Sclerosis (PRMS)^4^.

Glatiramer acetate (GA) was first launched as Copaxone^®^ in 1997 in US. GA is a random peptide copolymer consisted of four amino acids including glutamic acid, lysine, alanine, and tyrosine with customized ratios. The average molecular weight of GA is 5000–9000 daltons, approximately. It modulates the immune response by reducing CNS inflammation, demyelination and axonal loss^1,2,4^. GA is one of the most widely used medicines among disease-modifying therapies (DMTs)^2^. Subcutaneous administration (S.C.) of GA was reported to be equally efficient in reducing burden of disease based on MRI analysis in comparison to subcutaneous interferon (IFN) β-1a and IFNβ-1b. However, other beneficial outcomes of GA were supported by results of two decades treatment in a complementary study^1,2,5^.

Epidemiologic studies reveal that MS incidence and prevalence is increasing in women in their 30 s or even younger who are the most childbearing potential age group among women. According to international guidelines, most DMTs are either contraindicated or require noticeable cautions during pregnancy and lactation. Avonex^®^, Rebif^®^ ,

Betaseron^®^ , Fingolimod, Natalizumab and daclizumab are all DMTs placed in “Category C”. However, Mitoxantrone is in “Category D” and would cause congenital abnormalities such as delayed growth and premature delivery according to animal studies. Teriflunomide is a Category X drug that is contraindicated both before and during pregnancy. Steroids administration including prednisolone and methyl prednisolone in the first trimester may cause congenital fissure of the hard palate and low weight of newborns. Nevertheless, GA is the only DMT that is approved by in US FDA as “Category B” drug which means it is preferably trusted to be safe during pregnancy and lactation^1,6,7^.

Safety data collected from GA clinical trials represent that this medicine is well tolerated in adults. It shows relatively desirable side-effects even during long-term administration^3^. Injection-site reactions were seen to be the main adverse reactions of GA administration^3,4^. About 60% of patients, who are receiving GA subcutaneously, would normally experience undesirable localized loss of fat tissue, on-site nodules, bruises, pains, inflammation and dermatitis. These adverse effects would happen due to fast release of GA in subcutaneous area. Sometimes the intensity of these side effects would lead to treatment discontinuation^1–4^. All in all, chronic parenteral administration might lead to less patient compliance and therapy adherence. Therefore, developing a novel drug delivery system for GA is highly needed^4^.

Alternative methods of administration have yet to be proved to be efficient in MS^4^. Filippi et al. reported that orally administered GA did not affect relapse rate or other clinical MRI parameters in clinical study while Teitelbaum et al. claim that oral GA might cause immunomodulation in preclinical animal studies^8,9^.

Buccal administration averts hepatic metabolism and gastrointestinal degradation and provides a charming alternative to oral administration. However, the buccal mucosa is not an idea absorbent organ so that drug absorption seems somehow problematic more specifically in case of protein and peptides. Further obstacles in buccal delivery of GA are drug stability and formulation palatability. On the other hand, GA tends to become adhesive and forms clots which is not desirable in formulation of buccal tablets^4^.

There is a fact that a large number of patients and clinicians have experienced more persistent clinical efficiency with injectable DMTs so that they will show less tendency toward switching to oral drugs. This is normally because; less long term safety data are available for newly launched products. So that, it might be assumed that parenteral DMTs still possess top place in MS management. However, additional attempts such as autoinjector application and manufacturing of novel drug delivery systems are inevitable in order to improve patient adherence to injectable DMTs^10^.

GA is a first-line peptide based injectable DMT and will show low bioavailability in oral administration. GA has a very short half-life so that repeated high doses administration within short time intervals are required in clinical protocols. These properties would potentiate injection-site reactions and decrease patient compliance. However, GA is assumed as a prospecting medicine in MS management due to its favorable safety profile. So that, designing a novel drug delivery system is required in order to meet the unmet needs mentioned above.

Initial burst release of nearly 20–50% is known as one of the major challenges in designing protein-encapsulated microparticles (Yeo & Park, 2004). Therefore, we decided to apply customized emulsion methods (Wu et al., 2008) using PLGA, chitosan (CH) and alginate (AL) to bypass this bottleneck by retarding the initial burst release and achieving a controlled release pattern. We also performed a comparative in-vivo safety and efficacy study of GA-CH-AL hydrogel microparticles and Iranian GA brand generic formulation of Copamer^®^ (20 mg/ml) in Black 6 mice in EAE animal model.

## Materials and methods

### Materials

Glatiramer acetate (GA) was purchased from Tofigh Daru Company (Iran). Resomer^®^ Poly(D,L,-latide-co-glycolide) RG 502, RG 503, RG 502H, Complete Freund’s adjuvant, D-α-Tocopherol succinate, Pertussis toxin ( PTX) was obtained from Sigma-Aldrich (Germany). Partially hydrolyzed Polyvinyl alcohol (MW 30 k, 70 k), methylene chloride and tween 80^®^ were prepared from Merck (Germany). Chitosan (≥ 80% deacetylation, Mw 80 k) was obtained from Yuhuan Oceanic Biochemistry Co. Ltd. (China). Alginate (6 mPS for 1% at 25 °C) was procured from Shanghai Chemical Reagent Company of Chinese Medicine. Myelin oligodendrocyte glycoprotein (MOG 55–35) was bought from GL Biochem Co Ltd (China). Heat-killed Mycobacterium Tuberculosis strain H37RA was obtained from Difco (US). Sodium alginate, SMG, UP LVG and UP MVM were purchased from FMC (US). EAE INDUCTION kit- EK2110 was ordered from Hooke laboratories (US). 2, 4, 6 Trinitrobenzenesulfonic acid 5% w/v in methanol (TNBSA) was prepared from Thermo Scientific (US).

### Preparation of GA-hydrogel-microparticles

In present study, double emulsion method was used to synthesis microparticles. Internal and external aqueous phases contain glatiramer acetate, vitamin E succinate, polyvinyl alcohol (MW 30,70 k) and Tween 80. Meanwhile organic phase was a combination of methylene chloride and ethanol. Biodegradable polymers including sodium alginate 1% dissolved in internal aqueous phase. Aqueous chitosan solution 2% was added to external aqueous phase. Poly (D, L, and-latide-co-glycolide) Resomer^®^ (RG 502, RG 503, RG 502H) were dispersed in oil phase in different proportions as stated in Table 1. Prepared emulsions were homogenized by ultrasound probe (HT200 Heishler) with a frequency of 100 Hz and 90% power. Particles were then washed by phosphate buffer saline (PBS) pH 7.4. After removing free drug, particulate colloidal system were freeze dried by Alpha 1–4 LSC plus (Christ, Germany). Free polymers and drug including sodium alginate, chitosan and GA were removed per case using excessive washing ,centrifugation and PES (Polyethersulfone) and PTFE (Polytetrafluoroethylene) filters with 10–100 K MW cut-offs as described by literature^3,12,20,21^. Optimum freeze drying process was obtained when colloidal medium was first frozen at −43 °C for 3 h. Drying was followed by primary drying at −20 °C for 24 h, and secondary drying at 20 °C for 15 h. All lyophilized samples were kept in 2–8 °C and were reconstituted before use.

**Table 1:** Formulations of PLGA hydrogel microparticles (a: products contain only sodium alginate, b: products contain only chitosan, c: products contain only PLGA, d: products contain sodium alginate, chitosan and/or PLGA, e: poly (lactic-co-glycolic acid), f: Poly Vinyl Alcohol, g: Sodium Chloride).

Product code	GA1–5^a^	GC6–10^b^	GP11–15^c^	Gmix16–30^d^
**Aqueous phase**				
Glatiramer actate (GA) (mg)	10–40 mg	10–40 mg	10–40 mg	10–40 mg
**Oil phase**				
Methylene chloride	1-5 g	1-5 g	1-5 g	1-5 g
Alpha tocopherol (vitamin E) (mg)	0–30 mg	0–30 mg	0–30 mg	0–10 mg
**Polymer phase**				
Sodium alginate 1% (mg)	50–100 mg	0	0	10–60 mg
chitosan(mg)	0	10–150 mg	0	10–30 mg
PLGA^e^-506(mg)	0	0	50–300 mg	200–300 mg
PLGA-503(mg)	0	0	100–300 mg	150–250 mg
PLGA-502 h(mg)	0	0	50–200 mg	100–200 mg
**Others**				
PVA^f^ 35 k	0.5–2% (30 ml)	0.5–2% (30 ml)	0.5–2% (30 ml)	0.5–2% (30 ml)
PVA 70 k	0.5–2% (30 ml)	0.5–2% (30 ml)	0.5–2% (30 ml)	0.5–2% (30 ml)
NaCl^g^	0.25–0.9 mg	0.25–0.9 mg	0.25–0.9 mg	0.25–0.9 mg
**Results**				
Loading (%)	5–10%	7–15%	45–60%	35–60%
Particle size- d (0,5)- (µm)	10–30	15–35	40–55	25–35
Entrapment efficiency (EE %)	15–65%	60–70%	65–85%	75–95%
Sustained release profile	No	No	Yes	Yes

### Morphology studies by scanning electron microscope (SEM)

GA-hydrogel-microparticles were fixed overnight with glutaraldehyde 3% (Agar Scientific, UK) in PBS, pH = 7.2 (Thermo Scientific, USA). Samples were then washed 3 times by 0.1 M PBS, pH = 7.2. Dehydration was performed by ethanol, 25%, 50% and 70% for 20 min for each concentration followed by 15 min treatment with 96% and 99.9% ethanol and 20 min of treatment with hexamethyldisilazane (Merck, Germany). After an overnight drying in room temperature, samples mounted on a stub and were coated with gold by sputter coater (SCD004, Balzers Union, Germany). Gold coated samples were observed by Zeiss DSM 960A SEM.

### GA loading and entrapment efficiency (EE %) measurement

For preparation of 400 μg/ml glatiramer acetate stock solution, 4.8 mg of GA (with Assay ~ 87%, Water content % ~ 5.5%) was dissolved into 10 ml deionized water and then 7 ml of 0.1 M borate buffer (reaction buffer) was added. GA standard solutions including 20, 50,100,150 and 200 μg/ml were prepared by serial dilution from 400 μg/ml stock. All samples were prepared in triplicate (n = 3). GA assay was performed by UV–VIS (Ultra-Violet-Visible) spectroscopy (OPTIMA TOKYO: SP-3000 Plus, Japan) by TNBSA method in λ = 420 nm as previously described by Hadidi et al.^3,12,13^.

Entrapment efficiency (EE%) was also measured by a High pressure liquid chromatography (HPLC) with LC-P 100 pump, UV–Visible detector (Waters, US) and Develosil RP-C4 column. Mobile phase was a mixture of acetonitrile and trifluoroacetic acid, filtered through a 0.45 μm membrane filters and degassed by sonication. Deionized water was used as diluent. The flow rate of mobile phase was maintained at 1 ml/min. Detection was carried out by UV detector at 220 nm. Chromatograms were investigated for GA retention times at 9 min and the areas under curve (AUC) of the peaks of GA. All measurements were performed on the basis of calibration curve constructed for GA at 20, 50,100,150 and 200 μg/ml in PBS, pH = 7.4. Loading capacity and Entrapment efficiency (EE %) was calculated by Formula 1–4, respectively in order to double check the measurements.

- Formula 1: Loading capacity = ((Total amount of GA-Free GA)/Microparticle-hydrogel powder weight) * 100
- Formula 2: Loading capacity = ((Total amount of entrapped GA)/Microparticle-hydrogel powder weight) * 100
- Formula 3: Entrapment efficiency (EE %) = ((Total amount of GA-Free GA)/ Total amount of GA) * 100
- Formula 4: Entrapment efficiency (EE %) = (Total amount of entrapped GA)/ (Total amount of GA) * 100

### In vitro drug release study

To perform in-vitro release study, 50 mg of freeze dried powder was added to glass vials containing 20 ml of PBS buffer , pH = 7.4 and sodium azide (0.05%). Then 0.5 ml samples were removed at 0, 3, 6, 12, 18, 24 and 30 days and replaced with 0.5 ml of PBS buffer, pH = 7.4 to maintain sink condition. Samples were centrifuged at 10,000 rpm for 5 min. 50 μl of supernatant was diluted with 0.5 ml mobile phase. GA amount was investigated by HPLC method described in section "GA loading and entrapment efficiency (EE%) measurment". The cumulative amount of GA released during 30 days was measured according to calibration equation.

### Safety and Efficacy study in EAE animal model

All mice were purchased from animal department of Pasture Institute of Iran (Karaj, Iran). There were 3 EAE groups of 12 female C57BL/6 mice and one Non-EAE control group of 6 female C57BL/6 mice, all aged 7–14 weeks up to 25 g weight. Groups and dosing regimen in EAE model are summarized in Table S1. The recommendations in the ARRIVE guidelines and American Veterinary Medical Association (AVMA) Guidelines were followed. Mice were kept in specific pathogen free room with 25 ± 2 °C, RH (Relative Humidity) of 50%, 12 h light and 12 h dark with free access to food and water. Immunization was done under isoflurane anesthesia as follows. Antigen (MOG35-55) was first emulsified in Complete Freund’s adjuvant. The prepared emulsion was injected at maximum 3 sites and 0.1 mL/site (0.3 mL/mouse in total). Pertussis Toxin (PTX) was freshly diluted with cold PBS and was kept in ice bath for 2 h before injection. PTX was injected intra peritoneal (I.P.) at dose of 200 ng / mouse on day 0 and 1, under sterile conditions using a biosafety cabinet to induce autoimmune encephalitis^14–16^. Typically, EAE begins 9–14 days after immunization, with peak of disease 3–5 days after start of EAE induction. The peak maintains for 1–3 days, followed by partial recovery. Mice were typically monitored for 4 weeks, during which they were remained chronically paralyzed. Their weight and clinical symptoms were reported daily according to scoring system summarized in Table S2. Due to chronically paralysis of mice during this period, they were fed individually to stay alive. Special care was taken to reduce their mortality. It is reported that mice stress tends to greatly decrease EAE intensity, so that minimizing mouse sensitivity and stress were achieved by optimizing lab environmental conditions such as light, sounds, temperature and humidity. Appropriate mice handling and suitable S.C. injection were also found critical in successful EAE induction. It should be noted that equivalent GA doses was adjusted for different formulations. The EAE scoring plan is summarized in supplementary Table S2. It should be noted that all experimental protocols, methods and reporting system were performed as evaluated and approved by research ethics committee of Islamic Azad Tehran Medical Sciences University, Pharmacy and Pharmaceutical Branches Faculty with approval ID of IR.IAU.PS.REC.1398.118.

### Pathological studies

Mice enrolled in EAE model were euthanized at the end of 28 days. Demyelination and inflammatory reactions in brain and spinal cord tissues were also examined by LFB (Luxol fast blue) and H&E (Hematoxylin and Eosin) staining according to well established protocols^17,18^. Liver, kidney and skin samples from the mice injection site were also collected in all groups for further pathological studies by H&E staining.

## Results

### GA loading and entrapment efficiency (EE %) measurement

The GA entrapment efficiency (EE%) measurement shows that GA-hydrogel-microparticles contain a combination of PLGA, sodium alginate (1%), 0.5% PVA 35 k, alpha-Tocopherol and 1% chitosan were significantly higher in entrapment efficiency (EE%) (Table 1). This might be explained by electrostatic interactions and crosslinking occure du to positive and negative charges of mentioned ingredients. Particle size distribution was analyzed by Mastersizer 2000 (Malvern UK) and GA-hydrogel-microparticles diameter obtained through DLS method are shown as d (0, 5) in Table 1.

### Morphology studies by SEM

**Figure 1:**
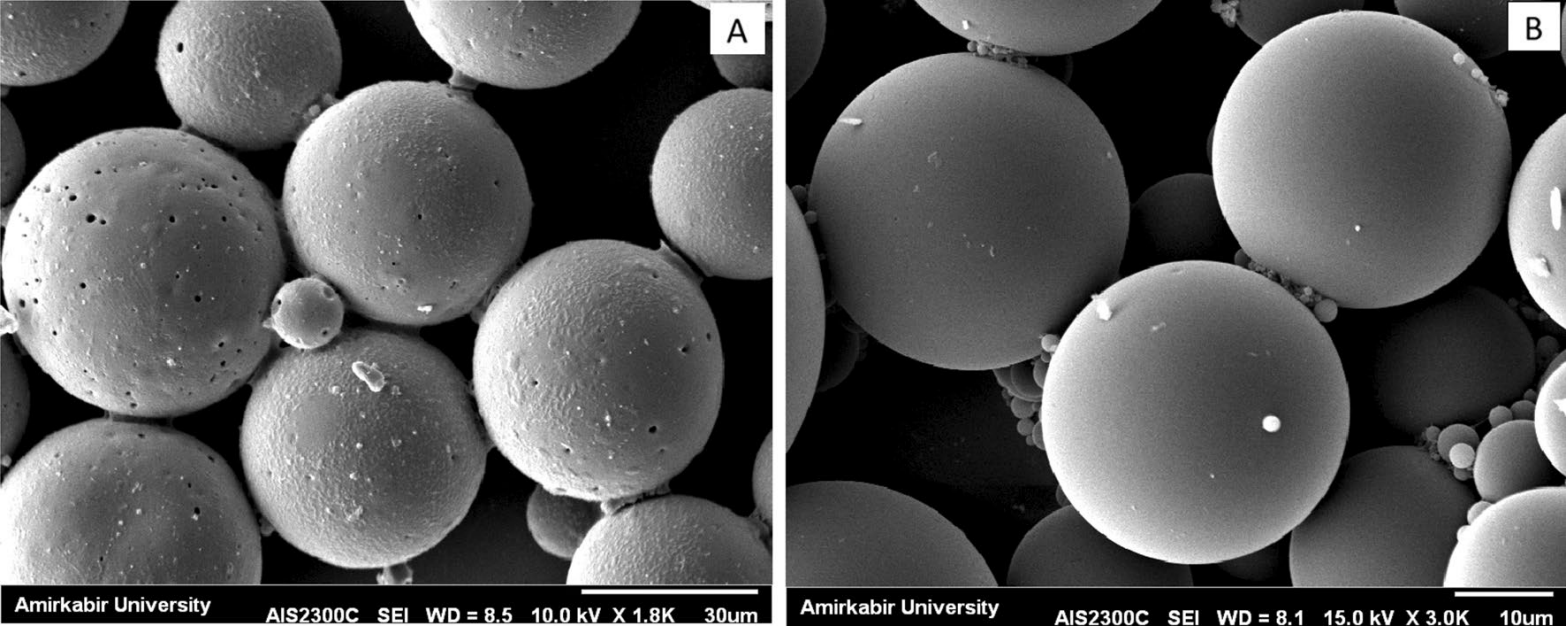
Morphology studies of conventional PLGA microparticles (**A**) and GA-PLGA-CH-AL hydrogel microparticles (GMIX22) (**B**) by SEM.

GA-PLGA-CH-AL (Glatiramer-PLGA-Chitosan-sodium Alginate) hydrogel microparticles displayed spherical, smooth, and non-aggregated micrographs under an optical microscope. To observe their surfaces and morphological properties more clearly, both conventional PLGA microparticles and GA-PLGA-CH-AL hydrogel microparticles were observed by SEM (Fig. 1A,B). Compared with conventional PLGA microparticles, GA-PLGA-CH-AL hydrogel microparticles showed significantly fewer surface pores and significantly lower surface roughness.

### In vitro drug release study

The release of GA was measured over a period of 30 days. Calibration curve was constructed in the range of 1–200 µg/mL. GA release was calculated by Eq. 1 where optical density (OD) was the difference at 420 and 700 nm and X is GA concentration (µg/mL). Results of GA release from GA-PLGA-CH-AL hydrogel microparticles suggest that GA/polymer ratio, polymer type, hydrophobic counter ion (alpha-Tocopherol), polymer charge and its molecular weight play roles in GA release pattern. Suitable peptide binding is required for sufficient GA loading. However, cross linkage of alpha-Tocopherol and polyamine molecules of applied polymers would prevent initial burst release and slow down the release pattern in the first 1–12 days. It should be noted that sampling was also done every 6 h during the first 72 h but data are not shown.

**Figure 2:**
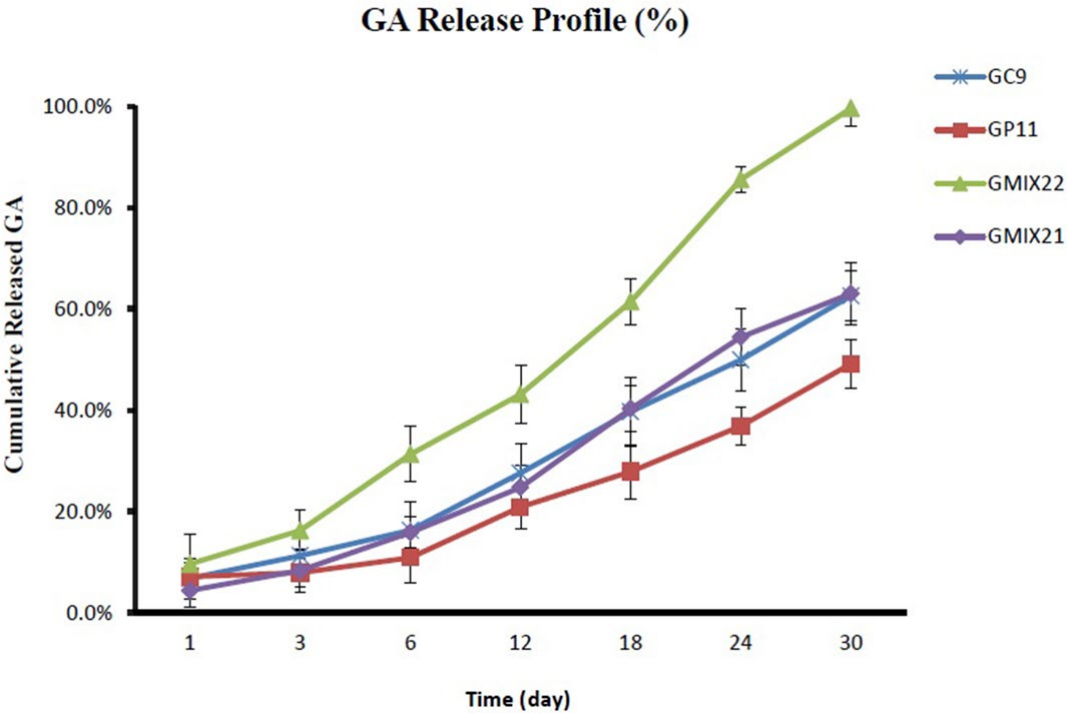
Comparative release profiles of GA (%) from formulation of GMIX21, GMIX22, GP9 and GP11.

It is assumed that multi-point ionic interaction between negative charge of polymers’ carboxyl groups and positive charge of GA lysine, directly influence binding capacity and release rate. Meanwhile, lower molecular weight polymers and optimized GA/polymer ratio, would provide better binding capacity, better control of initial burst release and more reasonable release rate during 30 days. As it is shown in Fig. 2, formulations containing different PLGA types and %PVA, with or without existence of counter ion, are totally distinguished in their retardants properties, maximum amount of GA release during study and GA% release in 1–12 days.

1 $${\text{OD }} = \, 0.0{\text{156X}} + 0.000{99},{\text{ R}}^{{2}} = \, 0.{9956}$$

### Efficacy study in EAE animal model

**Figure 3:**
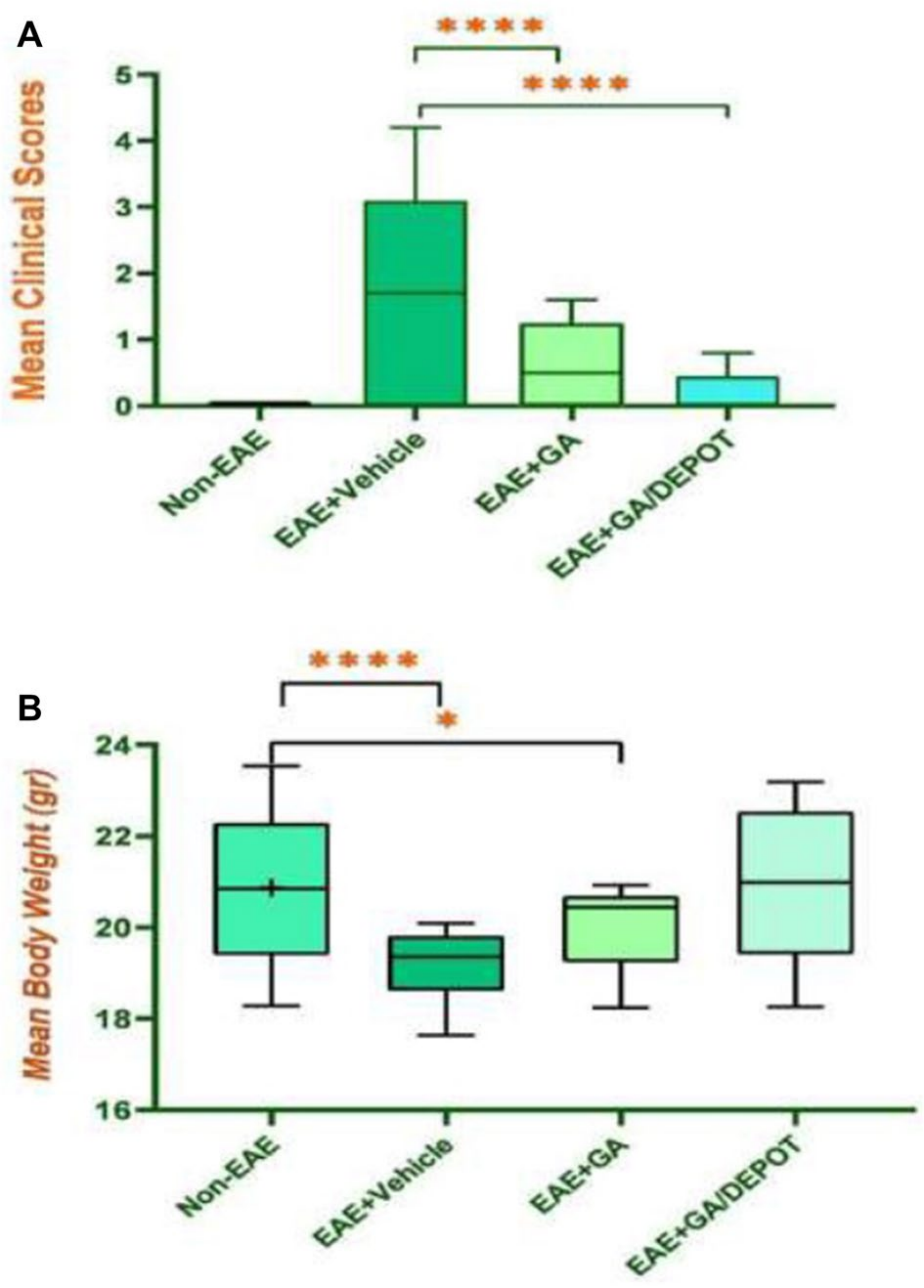
Comparative studies of mean clinical scores (**A**) and mean body weights (**B**) in four study groups over 28 days (p < 0.05 is shown by *, p < 0.01 is shown by ** and p < 0.001 is shown by ***) in 4 groups (GA-PLGA-CH-AL hydrogel microparticles (GMIX22) is briefly named as GA/Depot).

Experimental autoimmune encephalomyelitis (EAE) is an inflammatory autoimmune demyelinating disease which can be induced in laboratory animals. Therefore EAE would be assumed as standard animal model for researches related to autoimmune diseases such as multiples sclerosis (MS)^13,15^. Forty two female C57BL/6 mice were randomly enrolled in 4 groups. Table S1 summarizes mice groups, dosing regimen and dosing time in EAE mice model. EAE was induced by subcutaneous (S.C.) injection of MOG emulsion followed by intra peritoneal injection (I.P.) of PTX on day 0 and 48 h post MOG administration. EAE was assessed daily by clinical scoring of mice for 14 days post immunization according to a scale (0–5) (Table S2). Normal mice were scored as 0, while 1 was for limb tail, or hind limb weakness, 2 was for limp tail and hind limb weakness, 3 was for partial limb paralysis, 4 stand for complete hind limb paralysis and 5 was for moribund state during 14 days. Mean body weights were examined daily for 28 days post immunization with MOG. On day 28, mice were terminated and selected organs were extracted and fixed for pathological studies. Data were analyzed by ANNOVA followed by Dunnetts’ post-Hoc test, and Friedman test followed by Dunn’s multiple comparison test (p < 0.05 was considered as significant). As it is shown in Fig. 3A, GA-PLGA-CH-AL hydrogel microparticles (GMIX22) which is briefly named as GA/Depot (Fig. 3) significantly reduce mean clinical scores related to autoimmune encephalomyelitis development in comparison to EAE-vehicle group treated with vehicle and EAE-GA group that received Copamer^®^ as a conventional marketed product. On the other hand, GA-PLGA-CH-AL hydrogel microparticles (GMIX22) significantly prevent body weight loss in comparison to EAE-vehicle and EAE-GA group; however it was not significantly different from negative control (non-EAE) group (Fig. 3B). These outcomes confirms the improved efficacy of GA-PLGA-CH-AL hydrogel microparticles (GMIX22) in prevention of autoimmune encephalomyelitis in EAE mice model in comparison to conventional injection of GA.

### Safety study and pathological data

**Figure 4:**
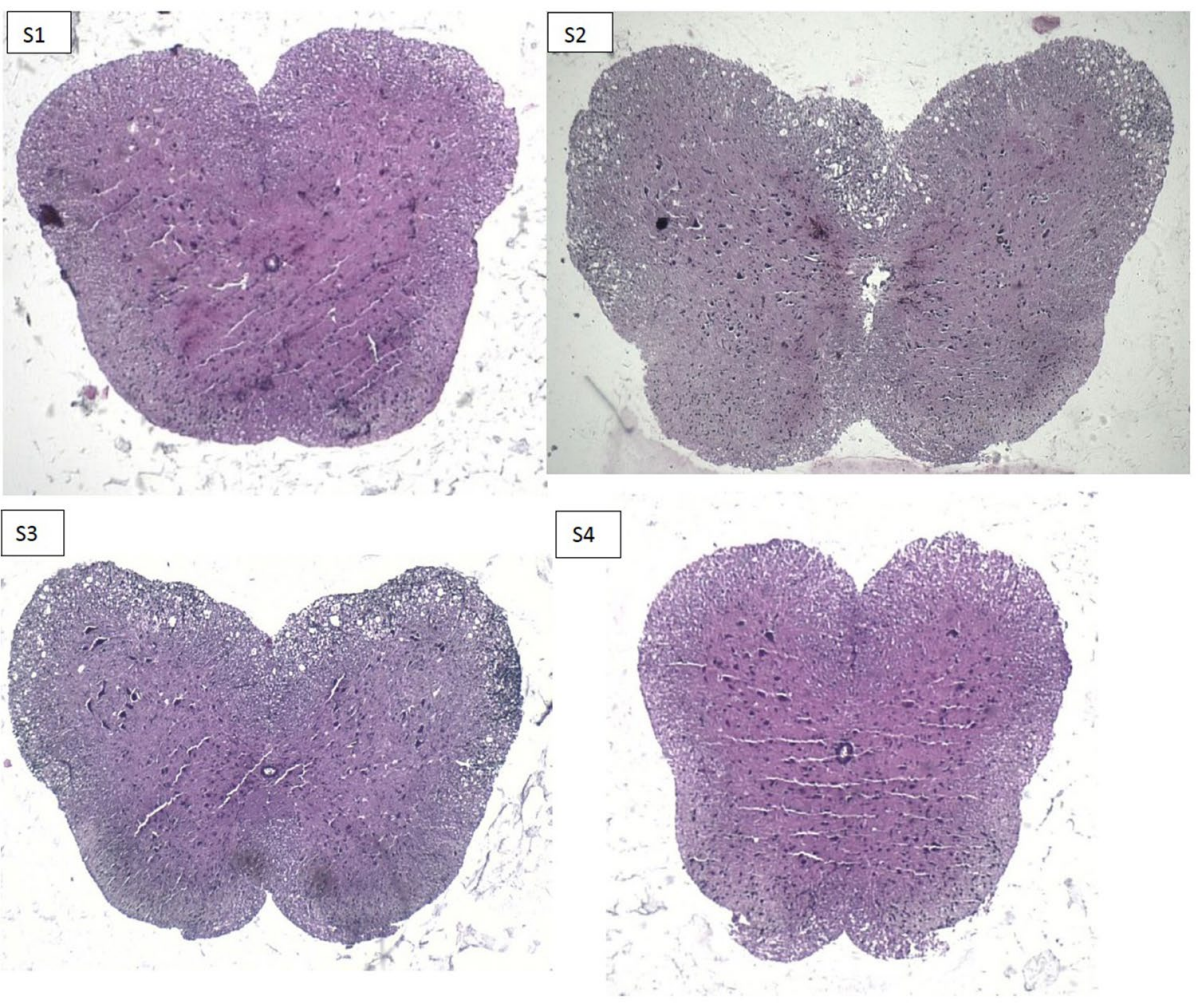
H&E stained pathological samples of Spinal cord End in: (S1) non-EAE group, (S2) EAE group, (S3) EAE-GA group, (S4) EAE-GA/Depot group.

**Figure 5:**
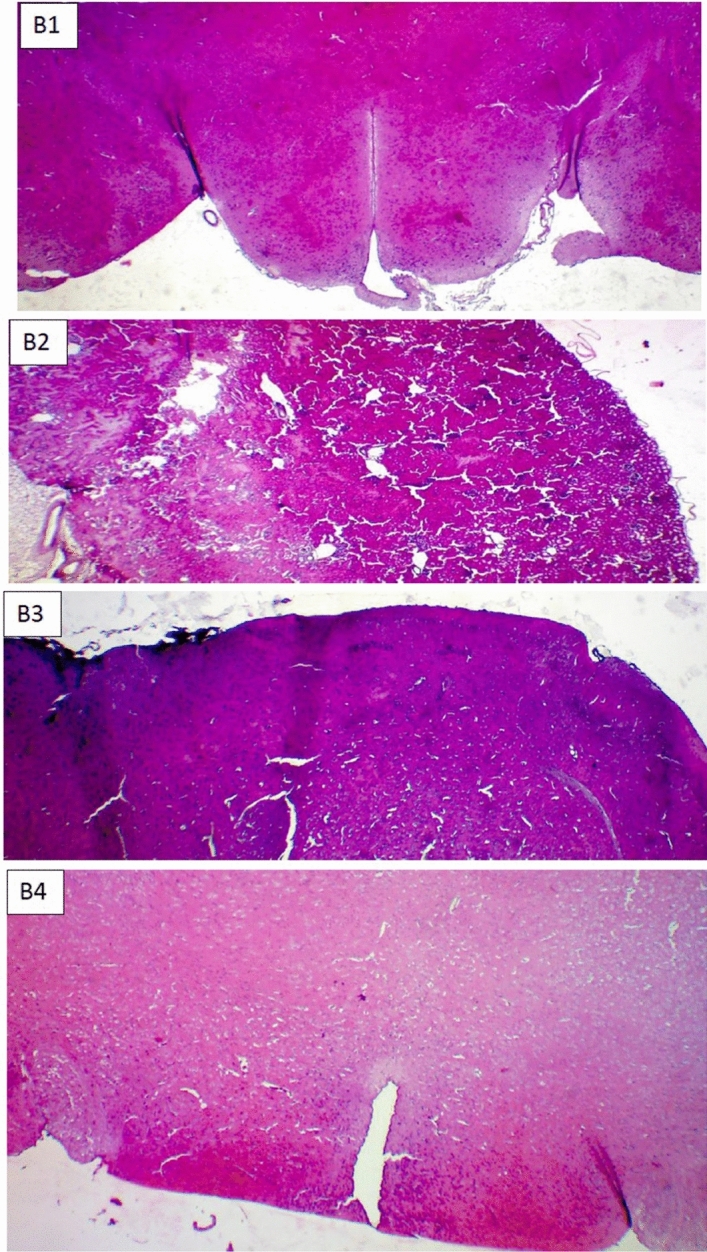
H&E stained pathological samples of Brain in: (B1) non-EAE group, (B2) EAE group, (B3) EAE-GA group, (B4) EAE-GA/Depot group.

**Figure 6:**
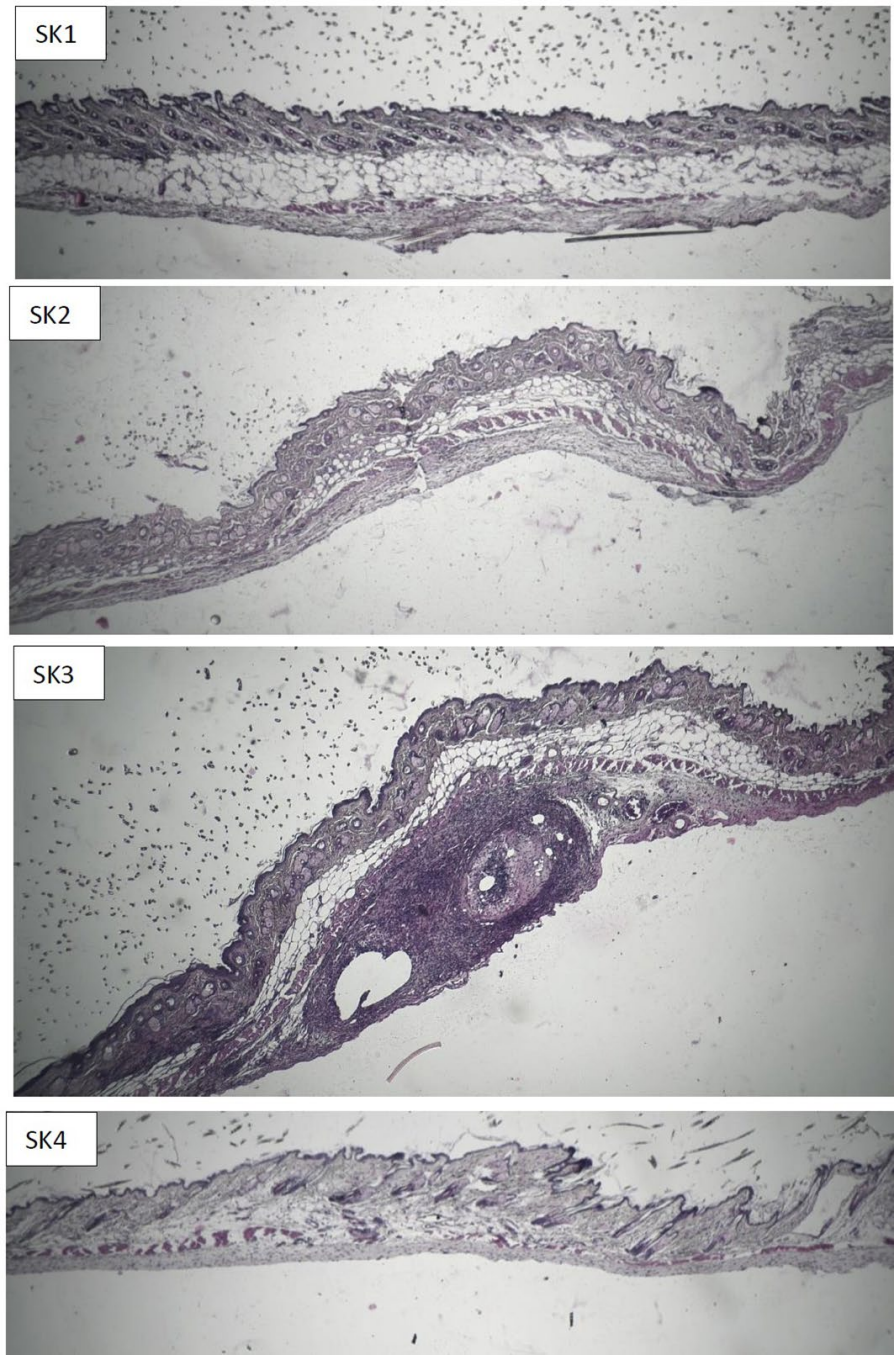
H&E stained pathological samples of Skin in: (SK1) non-EAE group, (SK2) EAE group, (SK3) EAE-GA group, (SK4) EAE-GA/Depot group.

**Figure 7:**
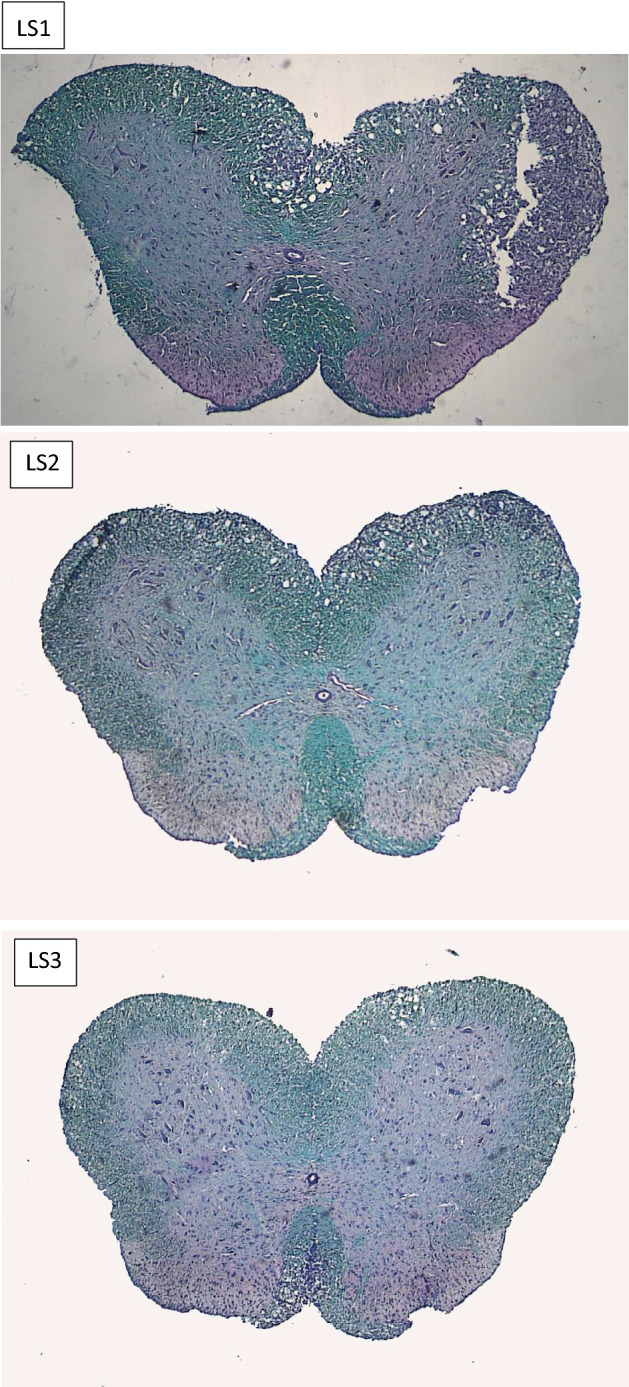
LFB stained pathological samples of first of spinal cord for demyelination comparison LS1 (EAE-first of spinal cord), LS2 (EAE-GA –end of spinal cord), LS3 (EAE-GA/Depot- end of spinal cord).

Pathological studies were also performed to investigate safety and efficacy of developed formulation. Mice were euthanized and their brain, spinal cord, liver, kidney tissues and injection site skin samples were collected in all groups to be stained by H& E and LFB (Figs. 4, 5, 6, 7). Figure S1 in supplementary file, H&E stained spinal cord (Figs. 4, 5) clearly show the presence of mononuclear inflammatory and inflammatory cells in the white matter in EAE-induced mice (Fig. 4, S2). This phenomenon confirms the validity of animal model when compared to non-EAE group (Fig. 4, S1). The outstanding point is that, brain and spinal cord shows mild to moderate presence of mononuclear inflammatory and inflammatory cells in the white matter of the spinal cord in comparison to GA treated mice (EAE-GA-group). However, Brian and spinal cord tissue in GA/Depot group was comparable to negative control (non-EAE) group. This observation confirms GA hydrogel microparticles efficacy in EAE animal model.

Infiltrations of hypodermic inflammatory cells and skin granulomatous are common adverse effects of GA conventional parenteral formulation at the injection site. This site reactions play key roles in poor patient compliance (Fig. 6-SK3). GA is an acidic medicine which its burst release in sub cutaneous area might cause irritation, necrosis and granulomatous in skin at the injection site. These adverse effects are omitted in the groups which were treated with GA/Depot hydrogel microparticles (Fig. 6-SK4) and the injection site is comparable to EAE-group and non-EAE group which received no GA injection (Fig. 6-SK1, SK2).

Figure 7 includes LFB staining used for detection of demyelination in spinal cord cross sectional samples in EAE-group, GA-group and GA/Depot group. As it is shown in Fig. 7, vast demyelination is obvious in LS1 but moderate demyelination in superior, medial and lateral segments of white matter are detectable in LS2. However, no sign of demyelination or damage is observed in GA/Depot group’ end part of spinal cord samples (Fig. 7, LS3).

## Discussion

Multiple sclerosis (MS) is a chronic immune mediated inflammatory disorder. Disease onset commonly occurs between the ages of 20–40 years of age with more prevalence in females. Parenteral drugs, i.e., interferon beta and Glatiramer acetate, are first-line therapeutics in MS, but long-term adherence to these injectable medicines is insufficient. Suboptimal adherence is of major concern in daily management of chronic disorders. It is said that injection-related non-adherence may accounts for up to two-thirds of missed injections and treatment failures so that suitable parenteral formulations and/or non- parenteral treatments are highly required to improve patient’s compliance and their adherence to MS treatment protocols^10^

The initial burst release of medicines and proteins are of great concern especially if they are highly toxic or this initial burst release might lead to adverse effects. Nodaway’s, scientists’ emphasis on development of micro and nano delivery systems is going to be switched to the development of novel hydrogel microparticles. Hydrogel microparticles are more desirable and advantageous than conventional hydrogel. Hydrogel microparticles would be customized to avoid burst release or achieve either prolonged or rapid drug release through conjugation with hydrophilic polymers. Moreover, this modification is a simple method and does not cause significant change in size and entrapment efficiency^19^. Drug loading in hydrogel microparticles would be either through post loading or in situ-loading. If post-loading method is applied, drug release is carried out by diffusion and swelling of hydrogel microparticles. But when in-situ drug loading is used, drug release can be determined by diffusion, hydrogel swelling and drug–polymer interactions^19^.

Hydrogels microparticles could be applied in preparation of solid dosage forms, imaging, tissue engineering, cell immobilizing and stimuli responsive polymeric drug delivery systems. Acrylic acid (AA), poly (N- isopropylacrylamide) (PNIPAAm), PVA, PEG (Poly Ethylene Glycol ) and chitosan are the most popular polymers which might avoid degradation of acid labile molecules such as protein and enzymes in controlled release stimuli responsive hydrogels microparticles (20). There are several methods to synthesize PLGA particles. Particles size, entrapment efficiency, release profile, and stability of drugs in the polymer matrix are highly dependent on particles preparation method. The most commonly used techniques for encapsulation of proteins and peptides into PLGA particles are spray-drying, double emulsion, solvent evaporation and Nano precipitation^19^. Spray-dried PLGA microspheres were able to encapsulate deferoxamine (DFO), a highly water soluble drug. The inclusion of DFO microspheres into chitosan/alginate hydrogel provided an efficient drug delivery with high entrapped DFO in the hydrogel network. DFO’s sustained released pattern was mainly through diffusion mechanism^20^.

In this study we use double emulsion/solvent evaporation technique. High yields, high encapsulation efficiency and suitability for hydrophilic-hydrophobic active molecules are main advantages of double emulsion (W/O/W) method. However, it was decided to modify conventional double emulsion method in order overcome particles size, surface porosity and release rate problems. Our investigation reveals that the duration and intensity of sonication could be effective in size distribution of the PLGA particles. The mean particles size and poly dispersity index were decreased when the sonication time was increased. Researchers claimed that organic solvent and hydrophobic interaction between PLGA and proteins might influence protein stability and could lead to some instability in the form of protein unfolding and aggregation. Previous studies have shown that proteins with positive charges could interact with the PLGA degradation and prevents fast drug release^20,21^. Physicochemical characterization of GA-hydrogel microprticles, conform all mentioned theories.

Mannitol and Poly vinyl alcohol (PVA) were used as protein stabilizer and lyoprotectant. Tween 80 was applied to prevent unalterable GA aggregation during preparation of PLGA micropartucles. Amino polysaccharide basis of chitosan, its biodegradability, good adhesion and cost effectiveness were important factors to achieve optimum GA loading and avoiding initial GA burst release. Alginate was also applied due to its hydrophilic nature, biocompatibility and biodegradability properties. Alginate was consumed as a gelling agent and physical cross linker in GA-hydrogel microprticles. Results indicated that the entrapment efficiency and release pattern were obviously influenced by alginate concentration and its viscosity in internal aqueous phase.

Finally, EAE animal model was performed in C57BL/6 mice to compare GA-hydrogel-microparticles and conventional GA parenteral formulation (Copamer^®^) in controlling clinical symptoms of MS attacks. Our purpose was to assess how this new formulation would fulfill the unmet needs among current Copamer^®^ users. Our in-vivo study demonstrated that GA hydrogel microparticles’ effect on disease symptoms and clinical observations were significantly better than Copamer^®^. Our pathological study also suggests that slow release of GA from GA hydrogel microparticles has a potential beneficial effect in in avoidance of infiltrations of hypodermic inflammatory cells and granulomatous in injection site. Other potential advantages achieved in this research would be summarized as deterrence of hypodermic inflammatory cells infiltrations and granulomatous in injection site, diminution of inflammatory responses and demyelination in brain and spinal tissues, as well as avoidance of fatty liver and edema in kidney nephron tubules. More importantly, significant superiority to Copamer^®^ in prevention of encephalomyelitis related symptoms in MS-specific EAE animal would assumed to lead to more patient’s compliance and treatment adherence.

## Conclusion

Multiple sclerosis (MS) is a chronic immune mediated inflammatory disorder. Suboptimal adherence is of major concern in daily management of chronic disorders such as MS. In this study, a novel chitosan-PLGA (poly (lactic-co-glycolic acid)) hydrogel-microparticles containing GA (GA-hydrogel-microparticles) were synthesized to address these unmet needs. GA-hydrogel-microparticles showed a sustained release profile with high loading and entrapment efficiency. It is assumed that avoidance of GA initial burst release might be a possible strategy in minimizing site injection adverse effects after GA parenteral administration. In -vivo studies through EAE animal model demonstrated that GA-hydrogel-microparticles possess potential benefits in RRMS especially regarding safety and efficacy in comparison to GA conventional parenteral administration. Further studies on pharmacokinetic and pharmacodynamic of GA-hydrogel-microparticles are still required to thoroughly examine different aspects of this novel formulation.

## Supplementary Information

Supplementary Information.

## Data Availability

The datasets used and/or analyzed during the current study available from the corresponding author on reasonable request. Supplementary data is available by authors.
