# Supplementary material for: Preparation, characterization and in-vivo efficacy study of glatiramer acetate (GA)-hydrogel-microparticles as novel drug delivery system for GA in RRMS
Source: Sci Rep. 2022 Dec 21;12:22042. doi: 10.1038/s41598-022-26640-x (PMC9772231; doi:10.1038/s41598-022-26640-x)
Supplement: Supplementary file 1 — Supplementary Information. [file 41598_2022_26640_MOESM1_ESM.docx]

**Table S1. Groups and dosing regimen in EAE model**

| **Group** | **Test** | **N** | **Route** | | **Concentration**  **( mg/ml)** | **ml/mice/day** | **mg/mice/day** | **Dose timing** |
| --- | --- | --- | --- | --- | --- | --- | --- | --- |
| 1 | PBS | 12 | | S.C | N/A | 0.2 | N/A | Day 0-9 |
| 2 | GA/Depot (GA-CH-AL hydrogel- microparticles GMIX22 ) | 12 | | I.M |  | 0.2 |  | Day 0 & Day 1 |
| 3 | GA-Copamer®  (positive control) | 12 | | I.M | 20 | 0.2 | 5 | Day 0 & Day 1 |
| 4 | Non-EAE  (negative control) | 6 | | N/A | 0 | 0.2 | N/A | Day 0 & Day 1 |

**Table S2. The EAE clinical scoring plan at different stages**

| **Stage** | **Score** | **Clinical observation** |
| --- | --- | --- |
| **Onset and peak** | **0** | No clear changes in motor function compared to non-immunized mice.  When picked up by base of tail, the tail has tension and is erect. Hind legs are usually spread apart. When the mouse is walking, there is no gait or head tilting. |
|  | **0.5** | Tip of tail is limp: When picked up by base of tail, the tail has tension except for the tip. Muscle straining is felt in the tail, whenever the tail continues to move. |
|  | **1** | Limp tail: When picked up by base of tail, instead of being erect, the whole tail, drapes over finger. Hind legs are usually spread apart. No signals of tail movement are observed. |
|  | **1.5** | Limp tail and hind leg inhibition: When picked up by base of tail, the whole tail drapes over finger. When the mouse is dropped on a wire rack, at least one hind leg falls through consistently. Walking is very slightly wobbly. |
|  | **2** | Limp tail and weakness of hind legs: When picked up by base of tail, the legs are not spread apart, but held closer together. When the mouse is observed walking, it has a clearly apparent wobbly walk. One foot may have toes dragging, but the other leg has no apparent inhibitions of movement.  - OR -Mouse appears to be at score 0.0, but there are obvious signs of head tilting when the walk is observed. The balance is poor. |
|  | **2.5** | Limp tail and dragging of hind legs: Both hind legs have some movement, but both are dragging at the feet (mouse trips on hind feet).  - OR - No movement in one leg/completely dragging one leg, but movement in the other leg.  - OR - EAE severity becomes mild when picked up (as score 0.0-1.5), but there is a powerful head tilt that causes the mouse to sporadically fall over. |
|  | **3** | Limp tail and entire paralysis of hind legs (most prevalent).  - OR - Limp tail and almost entire paralysis of hind legs. One or both hind legs are able to paddle, but neither hind leg is able to move forward of the hind hip.  - OR - Limp tail with paralysis of one front and one hind leg.  - OR - ALL of: Severe head tilting, Walking only along the edges of the cage, Pushing against the cage wall, Spinning when picked up by base of tail. |
|  | **3.5** | Limp tail and complete paralysis of hind legs. In addition to:  Mouse is moving around the cage, but when placed on its side, is unable to right itself. Hind legs are together on one side of body.  - OR - Mouse is moving around the cage, but the hind quarters are flat like a pancake, giving the appearance of a hump in the front quarters of the mouse. |
|  | **4** | Limp tail, entire hind leg and partial front leg paralysis.  Mouse is minimally moving around the cage but appears alert and feeding.  Often euthanasia is suggested after the mouse scores 4.0 for 2 days. Although, with daily subcutaneous fluids most C57BL/6 mice may recover to 3.5 or 3.0, while SJL mice may fully recover even if they reach score 4.0 at the peak of disease. When the mouse is euthanized because of prevalent paralysis, a score of 5.0 is entered for that mouse for the rest of the experiment. |
|  | **4.5** | Entire hind and partial front leg paralysis, no movement around the cage. Mouse is not alert.  Mouse has minimal movement in the front legs. The mouse barely reacts to contact.  Euthanasia is suggested. When the mouse is euthanized because of prevalent paralysis, a score of 5.0 is entered for that mouse for the rest of the experiment. |
|  | **5** | Mouse is spontaneously rolling in the cage (euthanasia is suggested).  - OR - Mouse is found dead due to paralysis.  - OR - Mouse is euthanized due to severe paralysis. |
| **Recovery** | **0** | When held by the base of tail, tail is partially “hooked” and rigid, but tail makes entire rotations around the body axis (“helicopter”). Mouse is healthy. No signals of wobbling. |
|  | **0.5** | Mouse appears normal but tail is “hooked” and rigid. Tail does not make entire rotations around the body axis (“helicopter”). Mouse is healthy. No signals of wobbling. |
|  | **3** | Mouse is found on its side (as explained for score 3.5 above), but there is extreme hind leg movement. Mouse cannot walk.  - OR - Mouse has a wobbly walk (as explained for score 2.5 above), and is unable to take more than two steps without falling on its side. The mouse is unable to right itself.  - OR - Mouse has poor movement in the hind legs (as explained for score 2.5 above), and has partial front leg paralysis evidenced by head held lower than normal and mouse's inability to right itself when placed on its side. |
|  | **All other scores** | Subtract 0.5 from the score of all mice with either a rigid, “hooked” tail or pedaling of hind legs. |

**
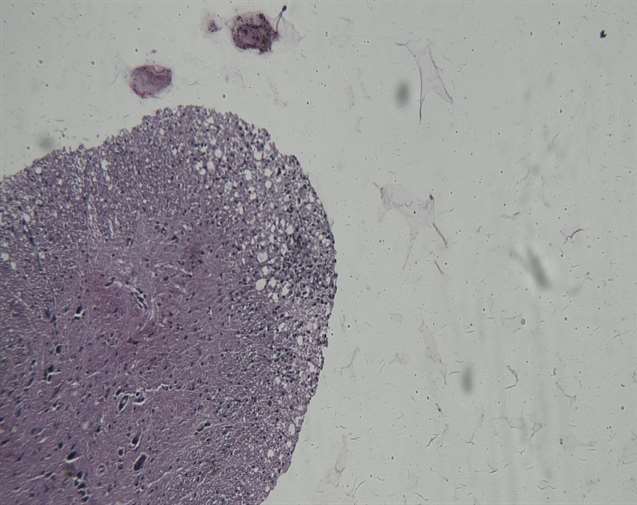

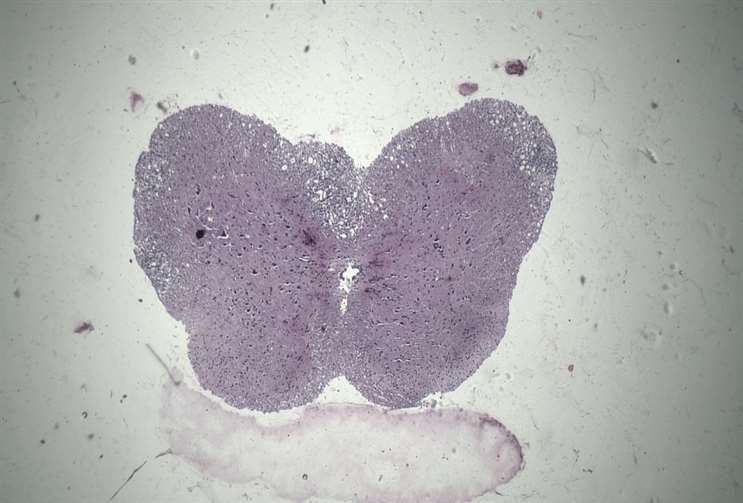
**

B

A

**
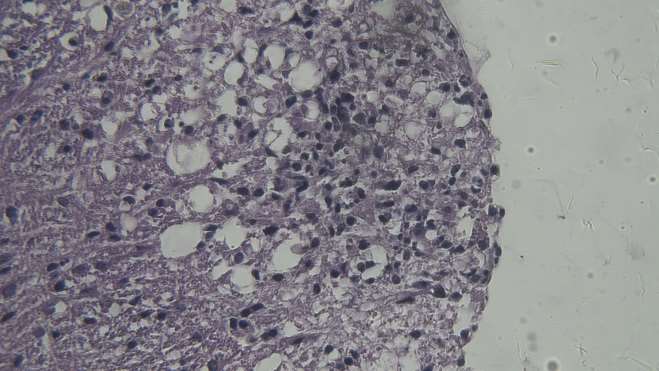
**

C

**Figure S1: *H&E stained pathological sample of Spinal cord End from EAE group A)4X,B)10X,C)40X***

**Table S3. List of abbreviation**

| GA | Glatiramer Acetate |
| --- | --- |
| RRMS | Relapsing Remitting Multiple Sclerosis |
| FDA | Food and Drug Administration |
| CNS | Central Nervous System |
| DMTs | Disease Modifying Therapies |
| PLGA | poly(lactic-co-glycolic acid) |
| EAE | Experimental Autoimmune Encephalomyelitis |
| H&E | Hematoxylin and Eosin |
| EE% | Entrapment Efficiency |
| MS | Multiple Sclerosis |
| RRMS | Relapsing-Remitting Multiple Sclerosis |
| SPMS | Secondary Progressive Multiple Sclerosis |
| PPMS | Primary Progressive Multiple Sclerosis |
| PRMS | Progressive Relapsing Multiple Sclerosis |
| S.C. | Subcutaneous administration |
| PBS | Phosphate Buffer Saline |
| PES | Polyethersulfone |
| PTFE | Polytetrafluoroethylene |
| HPLC | High pressure liquid chromatography |
| UV-VIS | Ultra-Violet-Visible |
| TNBSA | Trinitrobenzenesulfonic acid |
| AUC | areas under curve |
| RH | Relative Humidity |
| PTX | Pertussis Toxin |
| I.P. | Intra Peritoneal |
| PLGA | Poly (lactic-co-glycolic acid) |
| PVA | Poly Vinyl Alcohol |
| GA-PLGA-CH-AL | Glatiramer-PLGA-Chitosan-sodium Alginate |
| SEM | Scanning Electron Microscope |
| OD | Optical Density |
| AA | Acrylic acid |
| PNIPAAm | Poly (N- isopropylacrylamide |
| PEG | Poly Ethylene Glycol |
| DFO | deferoxamine |
